# Influence of Cutting Parameters on the Surface Quality of Two-Layer Sandwich Structures

**DOI:** 10.3390/ma13071664

**Published:** 2020-04-03

**Authors:** Elżbieta Doluk, Anna Rudawska, Józef Kuczmaszewski, Paweł Pieśko

**Affiliations:** Department of Mechanical Engineering, Lublin University of Technology, 20-388 Lublin, Poland; a.rudawska@pollub.pl (A.R.); j.kuczmaszewski@pollub.pl (J.K.); p.piesko@pollub.pl (P.P.)

**Keywords:** sandwich structure, milling, cutting parameters, aluminum alloy, CFRP

## Abstract

Hybrid sandwich structures are more and more widely used in many industries. This is mainly due to their good properties. One of the limitations regarding the use of sandwich structures is their difficult processing. Therefore, it seems reasonable to determine the influence of cutting parameters and machining configuration on the characteristic defect (phase) formed at the boundary of the materials forming a hybrid sandwich structure. This study investigates the effects of layer orientations during milling and machining parameters such as the cutting speed V_c_, the feed f_z_ and the cutting width a_e_. The study is conducted on a two-layer sandwich structure composed of two materials: 2024 aluminum alloy and epoxy-carbon composite with 60% of high-strength carbon fibers. A statistical analysis is performed using the Statistica program. The results show that the change in the cutting parameters has a greater impact on the formation of a defect on the surface of samples when the machining process starts on the side of the composite rather than the metal. The highest defect value is obtained for the milling from the composite layer when the process is performed with the following cutting parameters: V_c_ = 300 m/min, f_z_ = 0.08 mm/tooth, a_e_ = 5 mm.

## 1. Introduction

Along with a very dynamic development of industries related to machine design and maintenance, the use of innovative material solutions is increasing. It is necessary to search for materials that are lightweight and durable at the same time, due to increasingly strict requirements for heavily loaded structures. A composite sandwich structure an example of a modern construction materials used in the aviation industry [1,2,3]. 

One of the main reasons for the use of this type of structures is the need for lightweight and durable structures [4]. Hybrid layer structures (consisting of metal and polymer composite) have many advantages compared to analogous solid structures. Their main advantage is reduced weight and high strength properties. In addition, such structures are characterized by high relative flexural strength and stiffness, high corrosion resistance and low thermal conductivity [5,6]. These and other advantages of this type of material mean that sandwich structures are widely used in many industries, including aviation, automotive, railway, and transport. A typical sandwich structure consists of one or more outer layers and the core made of some other material [7]. Materials with good mechanical properties are usually used for the outer layers, while lightweight materials with lower strength properties are used as the core [6,7]. The role of the outer layers is to primarily carry tensile or compressive loads, while the core carries, transverse loads. In addition, the core increases the rigidity of the structure, separates the outer layers and improves damping properties [8,9,10]. Besides the type of materials composing the structure, the properties of a sandwich are also influenced by the structure of the core (e.g., foam core, homogeneous core and lattice core) [11]. The choice of layer materials primarily depends on the purpose of the sandwich, the availability of components and potential costs]. Metal and non-metallic materials are used as face skins. Stainless steel, aluminum alloys and titanium alloys are usually used as metal materials. Non-metallic faces are composite materials, in particular, carbon fiber-reinforced polymers (CFRP), glass fiber-reinforced polymers (GFRP) and aramid fiber-reinforced polymers (ARFP) [7]. An interesting and environmentally friendly solution are sandwich structures based on natural and recycled materials. Examples of such solutions can be materials resulting from the combination of particles of winter rapeseed stalks, geopolymer and basalt fibers [12]. Another example is the sandwich structure with a thermal insulation core of recycled polyurethane foam with the addition of winter wheat husk [13]. Due to their heterogeneous structure, hybrid sandwich structures are regarded as difficult-to-process engineering materials [14]. Consequently, they are usually made close to the final shape. Different properties of individual layers contribute to the formation of heterogeneous quality after processing. Different machinability between metal and composite means different mechanisms of material being removed. Lopresto et al. [15] have shown that the mechanism of chip formation in fibre-reinforced polymers is based on different phenomena than that occurring in metals. The machinability of fibre-reinforced polymers appears to be influenced by cutting parameters, tool material, tool geometry and fibre orientation. The problems occurring during the machining of this type of structures also include intensive wear of the cutting tools, loss of consistency between the composite fibers (debonding) and between the layers of a structure (delamination) or difficulties in maintaining the required cutting temperature [16,17,18]. Effective machining of hybrid sandwich structures must combine the features of metal and composite machining. A cutting process, conducted improperly, will not only result in in lower quality of the product, but it also lead to the exposure of composite fibers. Therefore, the result is increased susceptibility of the structure to the external environment (chemicals, moisture) [17,18,19,20]. The most widely used methods of machining composite sandwich structures are milling, drilling and cutting with abrasive water jet, diamond blades or band saws [21].

Despite the fact that hybrid sandwich structures are often used in many fields of the industry, their cutting conditions have not yet been sufficiently studied. Most scientists examine the failure damage of sandwiches, compare their properties with the properties of solids or reviews current trends in the applications for this type of materials. In addition, the recommendations for processing hybrid sandwich structures are usually based on the results obtained in the processing of fibre-reinforced polymers. The purpose of this study was to determine the effects of technological parameters and layer configurations on the surface quality of a hybrid sandwich structure after milling.

## 2. Materials and Methods 

In the present study a two-layer hybrid sandwich structure was tested. The materials forming the structure were: 2024 aluminum alloy (Al) and carbon epoxy composite (CFRP). Both materials had the form of 500 × 500 × 6 mm boards (length, width and thickness). Due to a high content of copper, 2024 aluminum alloy is characterized by high fatigue strength and low corrosion resistance. The main area of application for this material is the aviation industry – it is mainly used for aircraft structural components such as fuselages, wing skins, bulkheads or carriers. In addition, this alloy is also used for aircraft equipment, for example supports, safety railings, seat constructions or covers [22]. The chemical composition of the alloy is presented in Table 1. 

The other material was epoxy-carbon composite made of the CM-Preg TI02 20/1000 CP006 90 prepreg manufactured by the c-m-p gmbh company. The matrix in the composite was CP006 thermoplastic modified epoxy resin with very good impact properties. High-strength (HT) carbon fibers in the form of carbon fabric were used as reinforcement. The composite was manufactured by pressure-vacuum impregnation using the Scholz autoclave. The following composite curing parameters were applied: heating and cooling gradient 2 °C/min, curing 130 °C for 1 h, pressure 0.4 MPa, vacuum 0.09 MPa. The volume fraction of carbon fibers in the cured composite was about 60%. Table 2 contains selected properties of the epoxy-carbon composite used in the experiment. 

The sandwich structure was obtained by joining 2024 aluminum alloy with the epoxy-carbon composite. Surface preparation for the bonding process included: Preliminary degreasing of the boards with acetone, manual grinding with the Scotch-Brite 07447+ abrasive cloth, re-degreasing with acetone, Water Break Test, and the application of the 3M AC 130 solution to the joined surfaces. The boards were bonded using the two-part structural adhesive Scotch-Weld EC-9323 B/A (3M, USA, St. Paul, MN) mixed in a weight ratio of 100:27. The adhesive was applied as an even, thin layer on both surfaces. The bonding process involved placing the boards in a vacuum bag at a pressure of 0.01 MPa, keeping the materials for 24 h at room temperature and seasoning the bonded boards for 7 days in the air. The real thickness of the adhesive was 0.1 ± 0.02 mm.

**Figure 1:**
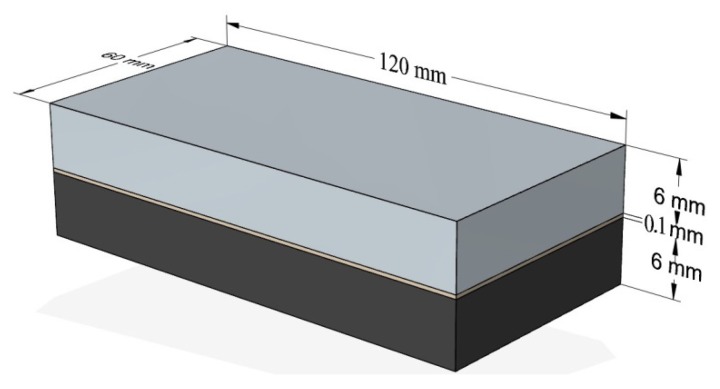
Shape and dimensions of sample.

**Figure 2:**
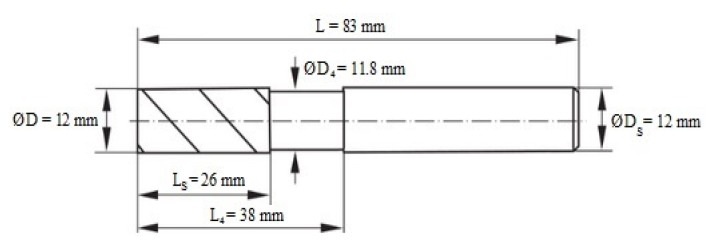
Shape and dimensions of tool [26].

The samples were cut and shaped into the size of 120 × 60 × 12 mm for length, width, and thickness, respectively. The samples were cut using the COMBO portal saw from Eckert AS at the following parameters: the cutting height H = 3 mm, the cutting speed V_c_ = 200 mm/min, the pressure of the abrasive jet P = 300 MPa, the abrasive expenditure Q = 0.5 kg/min. Figure 1 shows the schematic design of the tested sample. The processing of the bonded and cut samples involved peripheral milling (edging) using a universal tool dedicated to both aluminum alloy and polymer composite machining. The tool was a double-edged monolithic end mill with a helix angle λ = 45° (Garant, Germany, Munich). The shape and detailed dimensions of the tool are presented in Figure 2.

Machining was carried out on the FV-580A vertical machining center using variable technological parameters, such as cutting speed V_c_ (m/min), feed f_z_ (mm/tooth) and cutting width a_e_ (mm). 

**Figure 3:**
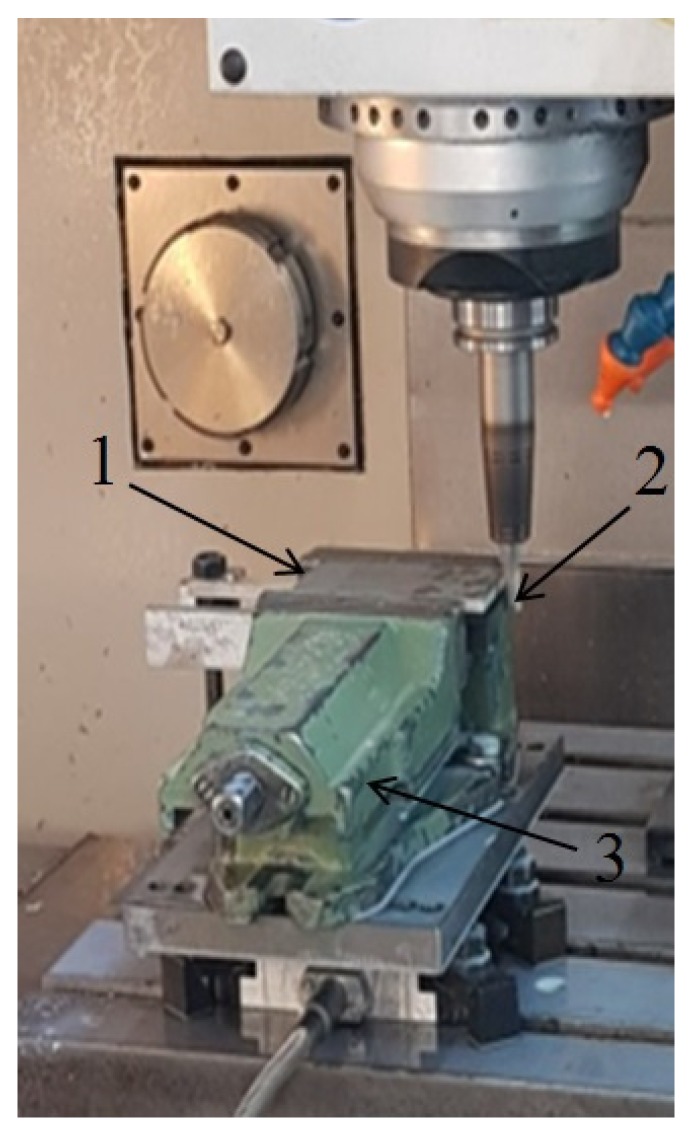
Milling of a sandwich structure: 1. sample, 2. tool, 3. machine vice.

**Figure 4:**
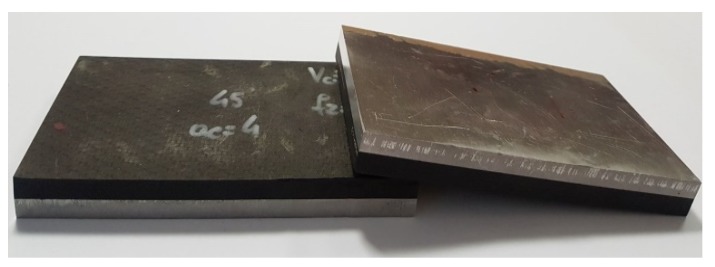
Samples after milling.

Figure 3 presents the milling process for one of the samples and Figure 4 shows the samples after processing. Table 3 lists the technological parameters that were adopted during processing. The technological parameters were selected based on the recommendations of the producer of the milling tool and the authors’ experience. The milling process was carried out for two configurations of sample layer arrangement: milling from the aluminum alloy layer (Al/CFRP) and milling from the composite layer (CFRP/Al). There were 10 samples after the abrasive cutting process: Five samples were milled in the Al/CFRP configuration and 5 samples were milled in the CFRP/Al configuration. The short side of the structure (60 mm) was processed. Each sample was milled on both sides – the experiment was repeated twice. 

**Figure 5:**
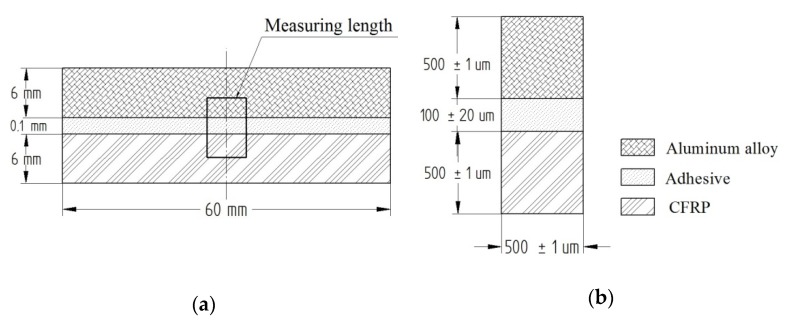
Dimensions of measuring length: (**a**) diagram of sample, (**b**) measuring length.

The defect formed at the boundary of the joined layers was examined using the VHX-500 microscope (Keyence, Japan, Osaka). The images were captured at a magnification of 500×. Figure 5 show the measuring length where the defect value was measured.

**Figure 6:**
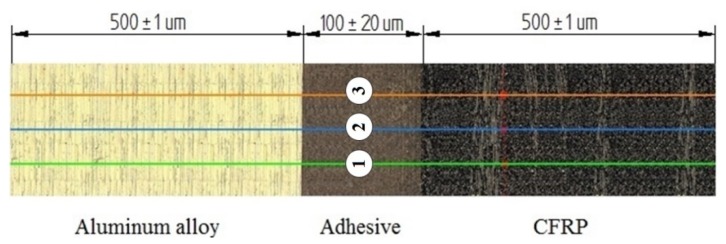
Measuring length with three measuring points.

Three measuring points were distinguished over the measuring length at which average values of the profile were measured (Figure 6). The obtained defect value for each of the tested samples was an arithmetic mean of 3 profile measurements at the designated measuring points. 

The work investigated the defect formation after the milling of a hybrid sandwich structure. The defect is defined as the difference between the heights of the cross profiles of unevenness that is formed on the metal and composite surfaces. This arises because the geometry to the workpiece is shaped by the tool feed rate. Properties of the materials composing the sandwich structure (density, stiffness, thermal conductivity, hardness, etc.) affect the cutting resistance in different ways. During the milling of the layer with higher density and better strength properties, the tool is pushed away from the material by the cutting forces. During the machining of the lower density layer, the cutting resistance decreases, hence the tool is drawn into the material. This results in the formation of a heterogeneous quality surface of the structure, which has a negative influence on its further operation.

**Figure 7:**
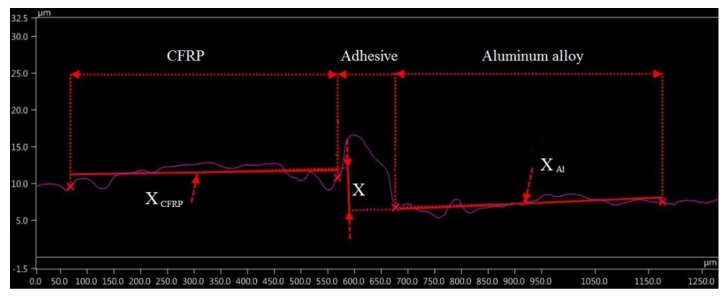
Method of determining the value of defect.

In order to define the value of the defect, the height of the cross profile of the unevenness (average value of 3 measurements) formed on the surface of the composite (X_CFRP_) and aluminum alloy (X_Al_) layers was determined (Figure 7). The distance between the heights was adopted as a defect after milling process (X) and was calculated by:

(1) $$X=\left|X_{CFRP}-X_{Al}\right|$$

where:

*X*  —the defect value (μm),
*X_CFRP_*  —the cross profile of the unevenness formed on the surface of the composite (μm),
*X_Al_*  —the cross profile of the unevenness formed on the surface of the aluminum alloy (μm).

**Figure 8:**
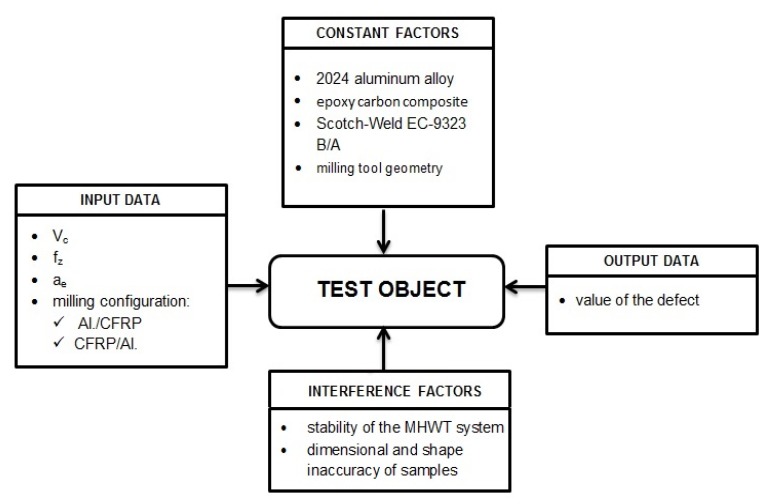
Research plan.

Figure 8 presents the schematic plan of the experiment. Materials, type of adhesive and geometry of the milling tool were the constant factors. The input data were variable processing parameters (V_c_, f_z_, a_e_) and milling configurations (Al/CFRP and CFRP/Al). The output data was the defect value. The stability of the Machine – Holder – Workpiece – Tool system (MHWT ) and the dimensional and shape inaccuracy of the samples were adopted as the interference factors.

A statistical analysis was performed, in order to examine the occurrence of statistically significant differences between the defect values that were obtained using different technological parameters and different configurations of the structure layers. The test results were statistically analyzed using the Statistica software. The following parameters were used in the statistical analysis: Correlation (determining an effective relationship between the variables) and multiple regression (describing the shape of this relationship). The assumed measure of the correlation between one variable and the total of the remaining variables was the multiple correlation factor (R). If R was close 1, the variables were in a linear relationship. The statistical analysis also included typical statistical tests. First, the Shapiro-Wilk test examining the distribution of quantitative variables was performed, then basic descriptive statistics were calculated for all considered variables. The samples not showing the normal distribution (W_α_ > W) were not subjected to a further statistical analysis. Next, the Fisher-Snedecor test was performed to check the homogeneity of the compared samples, followed by the parametric Student’s t-test for data showing the homogeneity of variance or the non-parametric Cochran-Cox test when the variance values of the compared samples were statistically significantly different. Table 4 gives the research hypotheses adopted for the purpose of the statistical analysis [27,28].

For the Fischer-Snedecor test, Student’s t-test and Cochran-Cox test, the H_0_ hypothesis was rejected in favor of the H_1_ hypothesis when the values of individual statistics (F, t and C) calculated for given samples were greater than the corresponding critical set (Table 4). Otherwise, there were no grounds for rejecting the H_0_ hypothesis. All these tests were performed at the significance level α = 0.05. 

## 3. Results 

### 3.1. Cutting Speed (V_c_) Effects on the Value of the Defect

**Figure 9:**
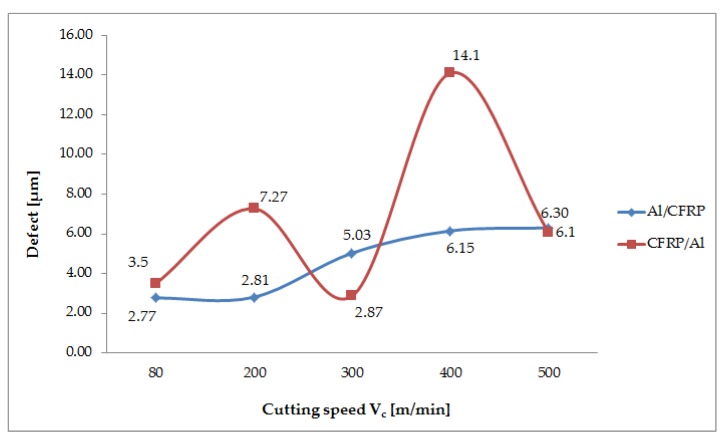
Effect of cutting speed change on value of defect.

The obtained value of the defect formed at variable cutting speeds for both configurations of the structure is shown in Figure 9. An analysis of the results demonstrates that, for the Al/CFRP configuration, an increase in cutting speed causes an increase in the defect value at the layer boundary. In this case, the higher cutting speed leads to deterioration of the quality of the machined surfaces. For the Al/CFRP configuration, the highest value of the defect (6.30 μm) is obtained for V_c_ = 500 m/min and the lowest (2.77 μm) for V_c_ = 80 m/min. When the process is carried out from the composite side (CFRP/Al), the trend in defect formation was irregular. The highest defect value (14.10 μm) is obtained for V_c_ = 400 m/min and the lowest (2.87 μm) for V_c_ = 300 m/min.

The difference between the maximum and minimum defect values obtained for the Al/CFRP configuration is nearly 57% and for the CFRP/Al configuration – it is 79%. The maximum defect of the CFRP/Al configuration is 55% greater than the maximum value obtained for the Al/CFRP configuration.

**Figure 10:**
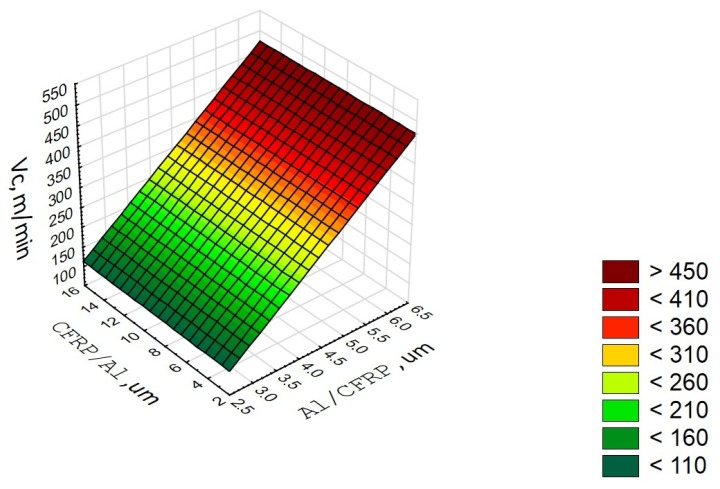
Diagram of correlation and multiple regression for V_c_ parameter.

The correlation factor R = 0.92 is significantly different from 0 (p = 0.14), which indicates a fairly strong correlation between the variable (V_c_) and the independent variables (defect values in the Al/CFRP and the CFRP/Al configurations). The determination factor is 0.71, which means that the adopted model accounts for 71% of the cutting speed variation (Figure 10).

The statistical analysis began with examining the normality of the results (Table 5). It is found that, for both configurations, the distributions of the considered variables do not differ significantly from the normal distribution. The statistical test results in relation to the defect values obtained at the variable cutting speed V_c_ are presented in Table 6 and Table 7. An analysis of the results demonstrates that, in the milling case, all results obtained for the tested cutting speeds show a homogeneity of variance. The results of the Student’s t-test indicate statistically significant differences between the V_c200_–V_c300_, V_c200_–V_c400_ and V_c200_–V_c500_ samples. At other cutting speeds, the value of the defect is not affected significantly. The statistical tests also show that the change in the cutting speed affects the defect results obtained in the milling of the CFRP/Al configuration. In all the compared samples, statistically similar results were obtained in three cases. Other samples show statistically significant differences at the a level of significance α = 0.05.

### 3.2. Feed (f_z_) Effects on the Value of the Defect

**Figure 11:**
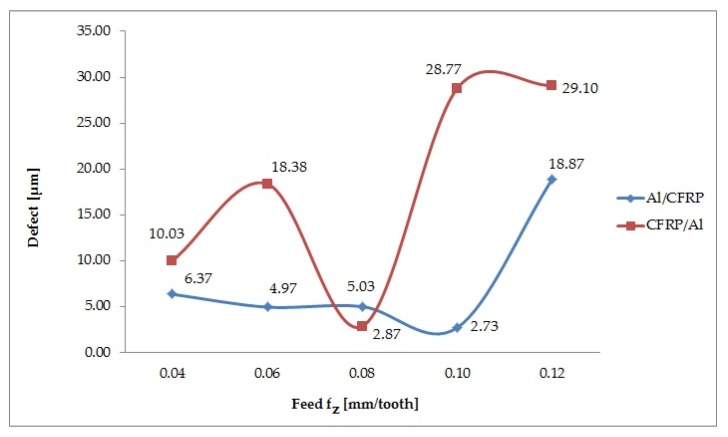
Effect of feed change on value of defect.

Figure 11 shows the defect value for the variable feed value. Examining the results obtained for the Al/CFRP configuration it can be observed that as the feed value is increased (to f_z_ = 0.10 mm/tooth the lowest tested value) the defect value decreases. The feed f_z_ = 0.12 mm/tooth causes a rapid increase to the maximum defect value (18.87 μm) and thus a deterioration of the surface quality. The lowest value (2.73 μm) is obtained at f_z_ = 0.10 mm/tooth. The defect results obtained after processing from the epoxy-carbon composite shown in Figure 11 are very irregular. It can be concluded that the lowest defect value (2.87 μm) and highest quality surfaces are obtained when f_z_ = 0.08 mm/tooth. The surface with the lowest surface quality (29.10 μm) is obtained during milling with the maximum feed value, i.e., f_z_ = 0.12 mm/tooth. The difference between the extremes for the Al/CFRP configuration is 86% and for the CFRP/Al configuration – 90%. The maximum defect value for the CFRP/Al configuration is about 35% higher than that obtained for the Al/CFRP configuration.

**Figure 12:**
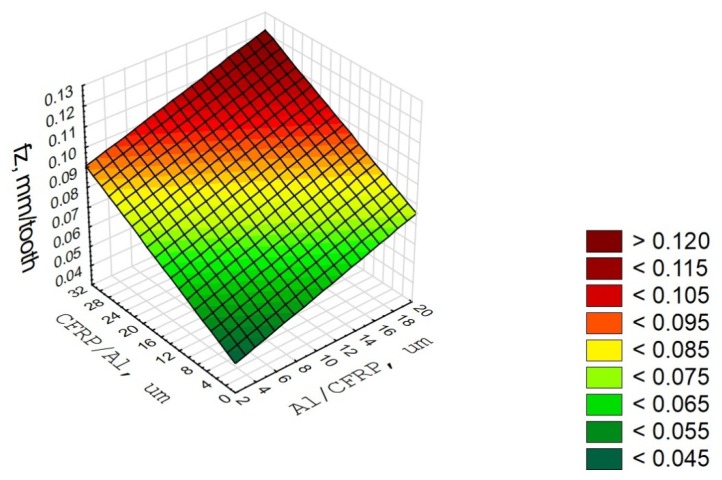
Diagram of correlation and multiple regression for f_z_ parameter.

The correlation factor R = 0.74 is significantly different from 0 (p = 0.05), which indicates a significantly correlation between the variable (f_z_) and the independent variables (defect values in the Al/CFRP and the CFRP/Al configuration). The determination factor is 0.71, which means that the adopted model accounts for 71% of the feed variation (Figure 12).

Results of the Shapiro-Wilk test are provided in Table 8. The results show that the distributions of most variables do not differ statistically to a significant extent from the normal distribution. A deviation is observed for the variable f_z_ = 0.12 mm/tooth for the CFRP/Al configuration. The values of this sample are not normally distributed and are not subjected to any further statistical analysis. Table 9 and Table 10 show the results of the statistical tests. 

The results show that for the Al/CFRP configuration half of the compared values are significantly different. It can be concluded the change in the f_z_ parameter for the considered arrangement of layers affects the surface quality of the samples after peripheral milling. The statistical analysis results show that all compared samples have exceeded the critical values. It can be deduced that the change in the f_z_ parameter significantly affects the value of the defect, formed at the boundary of the materials, comprising the sandwich structure.

### 3.3. Cutting width (a_e_) Effects on the Value of the Defect

**Figure 13:**
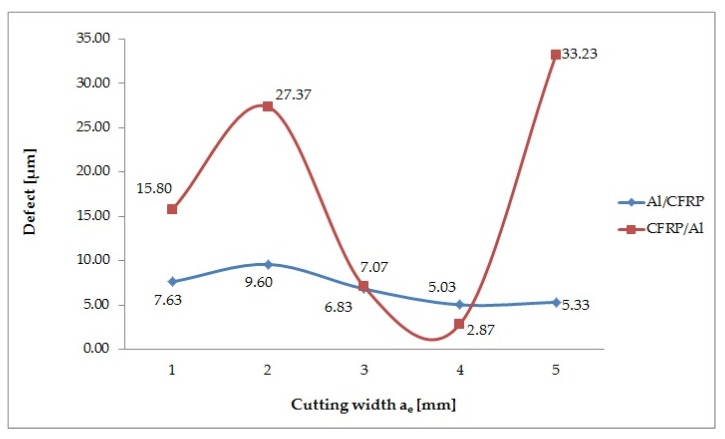
Effect of cutting width change on value of defect.

The influence of the cutting width a_e_ on the surface quality depending on the orientation of the layers in the sandwich structure is shown in Figure 13. In both configurations, an increase in the value of the defect can be observed when the cutting width is increased (a_e_ = 2 mm), followed by a decrease at a_e_ = 3 mm and a_e_ = 4 mm and a further increase in the maximum cutting width. During milling from the aluminum alloy layer (Al/CFRP), the highest value of the defect (9.60 μm) is obtained at the cutting width a_e_ = 2 mm, and the lowest (5.03 μm) at a_e_ = 4 mm. The highest defect value (33.23 μm) for the CFRP/Al configuration is obtained at a_e_ = 5 mm, and the lowest (2.87 μm) at a_e_ = 4 mm. The difference between the extreme and minimum defect values for the Al/CFRP configuration is nearly 48% and for the CFRP/Al configuration it is about 91%. The maximum defect value for the CFRP/Al configuration is about 71% greater than that obtained for the Al/CFRP configuration.

**Figure 14:**
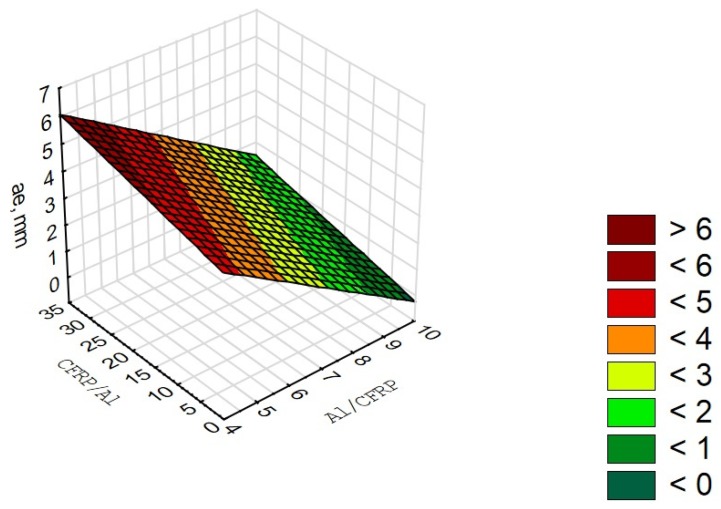
Diagram of correlation and multiple regression for a_e_ parameter.

The correlation factor R = 0.87 is significantly different from 0 (p = 0.02), which indicates a significant correlation between the variable (a_e_) and the independent variables (defect values in the Al/CFRP and the CFRP/Al configuration). The determination factor is 0.50, which means that the adopted model accounts for the 50% cutting width variation (Figure 14).

The Shapiro-Wilk test results are given in Table 11.

The defect values obtained during milling with the cutting width a_e_ = 1 mm for the Al/CFRP configuration show no normal distribution and are not subjected to further statistical tests. The remaining results do not differ significantly from the normal distribution. The effects of the statistical tests, performed for the Al/CFRP configuration, are compiled in Table 12.

Table 13 presents the results of statistical tests for the defect values obtained for the variable a_e_ during milling of the sandwich structure in the CFRP/Al configuration. All samples subjected to the Fisher-Snedecor test show statistically significant differences, while statistically similar results were obtained for samples that do not show the homogeneity of variance.

## 4. Discussion

Variable properties of the materials making up the sandwich structure cause damages after machining. One of them is the formation of a defect after milling, which makes it difficult to join sandwiches in large-size structures. Slamin et al. [29] and Uhlmann et al. [30] showed that a higher V_c_ during CFRP machining reduces the surface roughness parameter (Ra) and decreases the cutting forces. However, analysing the results obtained for the sandwich structure after milling in the Al/CFRP configuration demonstrates that the value of the defect increases with increasing V_c_ (V_c_ = 80 – 400 m/min) and then slightly decreases (V_c_ = 500 m/min). The trend of the defect for the CFRP/Al configuration is irregular – it is difficult to observe any relationship between defect formation and the occurrence of alternating high and low values. 

Gara and Tsoumarev [31] studied the effects of CFRP knurling and noticed the feed had the greatest impact on the surface roughness parameters. Azmi [32] and Boudelier [33] reached similar conclusions. They showed that the feed was the main parameter affecting the surface quality of CFRP after machining – an increase in the feed resulted in a decrease in the workpiece quality. With respect to the results, it can be observed that the maximum value of the defect for both tested configurations is obtained at the maximum feed value. For the Al/CFRP configuration the trend of the defect in the range of f_z_ = 0.04 – 0.08 mm/tooth remains at a similar level; at f_z_ = 0.10 mm/tooth it reaches the minimum value, and, finally, at f_z_ = 0.12 mm/tooth it rapidly increases (10 times) and reaches the maximum value. For the CFRP/Al configuration the minimum value of the defect is obtained at f_z_ = 0.08 mm/tooth. Increasing the feed to f_z_ = 0.10 mm/tooth and f_z_ = 0.12 mm/tooth results in a higher value of the defect on the surface of the sandwich structure.

The last parameter analysed under analysis is the cutting width (a_e_). The defect trend for Al/CFRP is more regular than that obtained for the CFRP/Al configuration - there are smaller differences in quality obtained for the same parameter. The values obtained for the Al/CFRP and CFRP/AL configurations range 5.03–9.60 µm, and 2.87–33.23 µm, respectively. The results of the CFRP/Al configuration do not allow prediction of the defect trend for a_e_. There is no research regarding the effect of the a_e_ parameter on the quality of the sandwich structure after milling, therefore, this problem should be investigated in further studies.

## 5. Conclusions

This paper investigated the impact of changing the technological parameters of machining on the surface quality of hybrid sandwich structures after peripheral milling. The experiments also focused on the effect of the orientation of layers in the structure. The results have demonstrated that the parameters that yield the lowest value of the defect are as follows: Al/CFRP - V_c_ = 300 m/min, f_z_ = 0.10 mm/tooth and a_e_ = 4 mm; for CFRP/Al - V_c_ = 300 m/min, f_z_ = 0.08 mm/tooth and a_e_ = 4 mm. Therefore, these parameters make it possible to obtain the highest surface quality. An analysis of the orientation of layers in the structure, during machining, revealed that the most favourable cutting conditions were ensured by the parameters that yielded the most similar value of the defect for both configurations. The values of the defect obtained for the same parameters in both configurations were then compared. On that basis, it was concluded that the parameters V_c_ = 500 m/min, f_z_ = 0.08 mm/tooth, a_e_ = 3 mm ensure the most similar surface quality of Al/CFRP and CFRP/Al. The most significant influence of the structure arrangement on the value of the defect (the highest difference between the configurations) was observed for the following parameters: V_c_ = 400 m/min, f_z_ = 0.10 mm/tooth, a_e_ = 5 mm.

Based on the results from the performed tests and statistical analyses, it can be concluded that in most cases the changes in the cutting parameters and layer orientation during milling had a significant impact on the value of the defect. The statistical analysis showed that milling in the CFRP/Al configuration had a more significant impact on the achieved defect for identical machining parameters, compared to the Al/CFRP configuration. Moreover, more samples processed in the CFRP/Al configuration compared to the Al/CFRP configuration showed significant statistical differences. Furthermore, the maximum defect values were achieved for this system of layers. No delamination was observed in the samples. In further research, an attempt will be made to determine the level of acceptability for defects occurring on the surface of sandwich structures after milling. Tests will be carried out to create indicators that determine the level of acceptability for such defects. 

## Figures and Tables

**Table 1:** Chemical composition of 2024 aluminum alloy [22].

Element	Si	Fe	Mg	Cu	Mn	Zn	Cr	Zr+Ti	Other	Al
Composition (%)	≤0.5	≤0.5	1.5	4.2	0.6	≤0.25	≤0.1	≤0.2	≤0.15	rest

**Table 2:** HT – carbon fiber/60 vol. % properties [23,24,25].

**Tensile strength 0°**	1100 MPa
**E-Modulus 0°**	70 GPa
**Flexural strength**	1050 MPa
**Flexural-Modulus 0°**	62 GPa
**ILSF**	70 MPa

**Table 3:** Technological parameters of milling.

Variable: V_c_ (m/min)	V_c_ = 80	f_z_ = 0.08	a_e_ = 4
	V_c_ = 200	f_z_ = 0.08	a_e_ = 4
	V_c_ = 300	f_z_ = 0.08	a_e_ = 4
	V_c_ = 400	f_z_ = 0.08	a_e_ = 4
	V_c_ = 500	f_z_ = 0.08	a_e_ = 4
Variable: f_z_ (mm/tooth)	V_c_ = 300	f_z_ = 0.04	a_e_ = 4
	V_c_ = 300	f_z_ = 0.06	a_e_ = 4
	V_c_ = 300	f_z_ = 0.08	a_e_ = 4
	V_c_ = 300	f_z_ = 0.10	a_e_ = 4
	V_c_ = 300	f_z_ = 0.12	a_e_ = 4
Variable: a_e_ (mm)	V_c_ = 300	f_z_ = 0.08	a_e_ = 1
	V_c_ = 300	f_z_ = 0.08	a_e_ = 2
	V_c_ = 300	f_z_ = 0.08	a_e_ = 3
	V_c_ = 300	f_z_ = 0.08	a_e_ = 4
	V_c_ = 300	f_z_ = 0.08	a_e_ = 5

**Table 4:** Research hypotheses and sets of critical values for Fisher-Snedecor test, Student’s t-test and Cochran-Cox test [27].

Test	Hypothesis	Set of Critical Values
Fisher-Snedecor	H_0_: $S_{1}^{2}=S_{2}^{2}S_{1}^{2}=S_{2}^{2}$ H_1_: $S_{1}^{2}\neq S_{2}^{2}$	W = <$F_{\infty };+\infty )$
Student’s t-test	H_0_: ${\overset{¯}{X}}_{1}={\overset{¯}{X}}_{2}$ ${\overset{¯}{X}}_{1}={\overset{¯}{X}}_{2}$H_1_: ${\overset{¯}{X}}_{1}\neq {\overset{¯}{X}}_{2}$	W = ($-\infty ;-t_{\propto })\;\cup <t_{\propto };+\infty )$
Cochran-Cox	H_0_: ${\overset{¯}{X}}_{1}={\overset{¯}{X}}_{2}$ ${\overset{¯}{X}}_{1}={\overset{¯}{X}}_{2}$H_1_: ${\overset{¯}{X}}_{1}\neq {\overset{¯}{X}}_{2}$	W = ($-\infty ;-C_{\propto })\;\cup <C_{\propto };+\infty )$

X—arithmetical mean; S^2^—variance.

**Table 5:** Shapiro-Wilk test results for V_c_ variable [27].

Variable	Configuration					
	Al/CFRP			CFRP/Al		
	W_α_	W	p	W_α_	W	p
V_c_ = 80	0.77	0.98	0.66	0.77	0.96	0.59
V_c_ = 200	0.77	0.95	0.58	0.77	0.87	0.30
V_c_ = 300	0.77	0.99	0.80	0.77	0.99	0.78
V_c_ = 400	0.77	0.99	0.96	0.77	0.99	0.95
V_c_ = 500	0.77	0.80	0.12	0.77	0.95	0.58

**Table 6:** Results of statistical analysis for V_c_ variable during milling from aluminum layer (Al/CFRP) [28].

Compared Parameters	Fischer-Snedecor Test			Student’s t-Test		
	F	F_α_ Critical Value	Result	t Statistics Value	t_α_ Critical Value	Result
	Statistics Value					
V_c80_–V_c200_	7.14	19.00	$S_{1}^{2}=S_{2}^{2}$	0.47	2.78	${\overset{¯}{X}}_{1}={\overset{¯}{X}}_{2}$
V_c80_–V_c300_	1.72	19.00	$S_{1}^{2}=S_{2}^{2}$	−1.76	2.78	${\overset{¯}{X}}_{1}={\overset{¯}{X}}_{2}$
V_c80_–V_c400_	1.46	19.00	$S_{1}^{2}=S_{2}^{2}$	−2.09	2.78	${\overset{¯}{X}}_{1}={\overset{¯}{X}}_{2}$
V_c80_–V_c500_	1.21	19.00	$S_{1}^{2}=S_{2}^{2}$	−2.35	2.78	${\overset{¯}{X}}_{1}={\overset{¯}{X}}_{2}$
V_c200_–V_c300_	4.16	19.00	$S_{1}^{2}=S_{2}^{2}$	−3.17	2.78	${\overset{¯}{X}}_{1}={\overset{¯}{X}}_{2}$
V_c200_–V_c400_	10.43	19.00	$S_{1}^{2}=S_{2}^{2}$	−2.98	2.78	${\overset{¯}{X}}_{1}\neq {\overset{¯}{X}}_{2}$
V_c200_–V_c500_	5.19	19.00	$S_{1}^{2}=S_{2}^{2}$	−3.73	2.78	${\overset{¯}{X}}_{1}\neq {\overset{¯}{X}}_{2}$
V_c300_–V_c4000_	2.51	19.00	$S_{1}^{2}=S_{2}^{2}$	−0.75	2.78	${\overset{¯}{X}}_{1}={\overset{¯}{X}}_{2}$
V_c300_–V_c500_	1.42	19.00	$S_{1}^{2}=S_{2}^{2}$	−0.82	2.78	${\overset{¯}{X}}_{1}={\overset{¯}{X}}_{2}$
V_c400_–V_c500_	1.76	19.00	$S_{1}^{2}=S_{2}^{2}$	0.06	2.78	${\overset{¯}{X}}_{1}={\overset{¯}{X}}_{2}$

**Table 7:** Results of statistical analysis for V_c_ variable during milling from composite layer (CFRP/Al) [28].

**Compared Parameters**	**Fischer-Snedecor Test**			**Student’s t-Test**		
	**F Statistics Value**	**F_α_ Critical Value**	**Result**	**t Statistics Value**	**t_α_ Critical Value**	**Result**
V_c80_–V_c200_	1.62	19.00	$S_{1}^{2}=S_{2}^{2}$	−3.13	2.78	${\overset{¯}{X}}_{1}\neq {\overset{¯}{X}}_{2}$
V_c80_–V_c400_	3.71	19.00	$S_{1}^{2}=S_{2}^{2}$	−5.17	2.78	${\overset{¯}{X}}_{1}\neq {\overset{¯}{X}}_{2}$
V_c80_–V_c500_	1.93	19.00	$S_{1}^{2}=S_{2}^{2}$	−2.23	2.78	${\overset{¯}{X}}_{1}={\overset{¯}{X}}_{2}$
V_c200_–V_c400_	6.01	19.00	$S_{1}^{2}=S_{2}^{2}$	−3.48	2.78	${\overset{¯}{X}}_{1}\neq {\overset{¯}{X}}_{2}$
V_c200_–V_c500_	1.19	19.00	$S_{1}^{2}=S_{2}^{2}$	6.10	2.78	${\overset{¯}{X}}_{1}\neq {\overset{¯}{X}}_{2}$
V_c400_–V_c500_	7.14	19.00	$S_{1}^{2}=S_{2}^{2}$	4.12	2.78	${\overset{¯}{X}}_{1}\neq {\overset{¯}{X}}_{2}$
**Compared Parameters**	**Fischer-Snedecor Test**			**Cochran Cox-Test**		
	**F Statistics Value**	**F_α_ Critical Value**	**Result**	**C Statistics Value**	**C_α_ Critical Value**	**Result**
V_c80_–V_c200_	42.32	19.00	$S_{1}^{2}>S_{2}^{2}$	2.60	4.30	${\overset{¯}{X}}_{1}={\overset{¯}{X}}_{2}$
V_c200_–V_c300_	26.11	19.00	$S_{1}^{2}>S_{2}^{2}$	4.75	4.30	${\overset{¯}{X}}_{1}\neq {\overset{¯}{X}}_{2}$
V_c300_–V_c400_	156.79	19.00	$S_{1}^{2}>S_{2}^{2}$	5.02	4.30	${\overset{¯}{X}}_{1}\neq {\overset{¯}{X}}_{2}$
V_c300_–V_c500_	21.95	19.00	$S_{1}^{2}>S_{2}^{2}$	3.36	4.30	${\overset{¯}{X}}_{1}={\overset{¯}{X}}_{2}$

**Table 8:** Shapiro-Wilk test results for f_z_ variable [27].

Variable	Configuration					
	Al/CFRP			CFRP/Al		
	W_α_	W	p	W_α_	W	p
f_z_ = 0.04	0.77	0.87	0.30	0.77	0.99	0.90
f_z_ = 0.06	0.77	0.99	0.96	0.77	0.96	0.64
f_z_ = 0.08	0.77	0.99	0.80	0.77	0.99	0.78
f_z_ = 0.10	0.77	0.88	0.33	0.77	0.78	0.77
f_z_ = 0.12	0.77	0.93	0.60	0.77	0.75	0.00

**Table 9:** Results of statistical analysis for f_z_ variable during milling from aluminum alloy layer (Al/CFRP) [28].

**Compared parameters**	**Fischer-Snedecor Test**			**Student’s t-Test**		
	**F Statistics Value**	**F_α_ Critical Value**	**Result**	**t Statistics Value**	**t_α_ Critical Value**	**Result**
f_z0.04_–f_z0.08_	17.84	19.00	$S_{1}^{2}=S_{2}^{2}$	1.66	2.77	${\overset{¯}{X}}_{1}={\overset{¯}{X}}_{2}$
f_z0.04_–f_z0.1_	3.32	19.00	$S_{1}^{2}=S_{2}^{2}$	9.42	2.77	${\overset{¯}{X}}_{1}\neq {\overset{¯}{X}}_{2}$
f_z0.06_–f_z0.08_	4.26	19.00	$S_{1}^{2}=S_{2}^{2}$	0.04	2.77	${\overset{¯}{X}}_{1}={\overset{¯}{X}}_{2}$
f_z0.06_–f_z0.12_	3.21	19.00	$S_{1}^{2}=S_{2}^{2}$	−5.28	2.77	${\overset{¯}{X}}_{1}\neq {\overset{¯}{X}}_{2}$
f_z0.08_–f_z0.1_	5.37	19.00	$S_{1}^{2}=S_{2}^{2}$	2.69	2.77	${\overset{¯}{X}}_{1}={\overset{¯}{X}}_{2}$
f_z0.08_–f_z0.12_	13.68	19.00	$S_{1}^{2}=S_{2}^{2}$	−5.82	2.77	${\overset{¯}{X}}_{1}\neq {\overset{¯}{X}}_{2}$
f_z0.1_–f_z0.12_	5.02	19.00	$S_{1}^{2}=S_{2}^{2}$	−6.77	2.77	${\overset{¯}{X}}_{1}\neq {\overset{¯}{X}}_{2}$
**Compared Parameters**	**Fischer-Snedecor Test**			**Cochran Cox-Test**		
	**F Statistics Value**	**F_α_ Critical Value**	**Result**	**C Statistics Value**	**C_α_ Critical Value**	**Result**
f_z0.04_–f_z0.06_	75.90	19.00	$S_{1}^{2}>S_{2}^{2}$	0.70	4.30	${\overset{¯}{X}}_{1}={\overset{¯}{X}}_{2}$
f_z0.04_–f_z0.12_	243.97	19.00	$S_{1}^{2}>S_{2}^{2}$	8.98	4.30	${\overset{¯}{X}}_{1}\neq {\overset{¯}{X}}_{2}$
f_z0.06_–f_z0.1_	22.85	19.00	$S_{1}^{2}>S_{2}^{2}$	1.10	4.30	${\overset{¯}{X}}_{1}={\overset{¯}{X}}_{2}$

**Table 10:** Results of statistical analysis for f_z_ variable during milling from composite layer (CFRP/Al) [28].

**Compared Parameters**	**Fischer-Snedecor Test**			**Student’s t-Test**		
	**F Statistics Value**	**F_α_ Critical Value**	**Result**	**t Statistics Value**	**t_α_ Critical Value**	**Result**
f_z0.04_–f_z0.06_	9.19	19.00	$S_{1}^{2}=S_{2}^{2}$	−8.22	2.77	${\overset{¯}{X}}_{1}\neq {\overset{¯}{X}}_{2}$
f_z0.04_–f_z0.08_	4.79	19.00	$S_{1}^{2}=S_{2}^{2}$	20.50	2.77	${\overset{¯}{X}}_{1}\neq {\overset{¯}{X}}_{2}$
f_z0.04_–f_z0.1_	5.09	19.00	$S_{1}^{2}=S_{2}^{2}$	−23.88	2.77	${\overset{¯}{X}}_{1}\neq {\overset{¯}{X}}_{2}$
f_z0.06_–f_z0.1_	1.81	19.00	$S_{1}^{2}=S_{2}^{2}$	−8.65	2.77	${\overset{¯}{X}}_{1}\neq {\overset{¯}{X}}_{2}$
**Compared Parameters**	**Fischer-Snedecor Test**			**Cochran Cox-Test**		
	**F Statistics Value**	**F_α_ Critical Value**	**Result**	**C Statistics Value**	**C_α_ Critical Value**	**Result**
f_z0.06_–f_z0.08_	44.00	19.00	$S_{1}^{2}>S_{2}^{2}$	12.99	4.30	${\overset{¯}{X}}_{1}\neq {\overset{¯}{X}}_{2}$
f_z0.08_–f_z0.1_	24.37	19.00	$S_{1}^{2}>S_{2}^{2}$	28.90	4.30	${\overset{¯}{X}}_{1}\neq {\overset{¯}{X}}_{2}$

**Table 11:** Shapiro-Wilk test results for a_e_ variable [27].

Variable	Configuration					
	Al/CFRP			CFRP/Al		
	W_α_	W	p	W_α_	W	p
a_e_ = 1	0.77	0.75	0.00	0.77	0.92	0.43
a_e_ = 2	0.77	0.93	0.49	0.77	0.99	0.92
a_e_ = 3	0.77	0.78	0.07	0.77	0.90	0.38
a_e_ = 4	0.77	0.99	0.80	0.77	0.99	0.86
a_e_ = 5	0.77	0.99	0.77	0.77	0.99	0.87

**Table 12:** Results of statistical analysis for a_e_ variable during milling from aluminum alloy layer (Al/CFRP) [28].

Compared Parameters	Fischer-Snedecor Test			Student’s t-Test		
	F Statistics Value	F_α_ Critical Value	Result	t Statistics Value	t_α_ Critical Value	Result
a_e2_–a_e3_	19.00	2.07	$S_{1}^{2}=S_{2}^{2}$	2.77	2.78	${\overset{¯}{X}}_{1}\neq {\overset{¯}{X}}_{2}$
a_e2_–a_e4_	19.00	1.90	$S_{1}^{2}=S_{2}^{2}$	2.77	4.72	${\overset{¯}{X}}_{1}\neq {\overset{¯}{X}}_{2}$
a_e2_–a_e5_	19.00	6.02	$S_{1}^{2}=S_{2}^{2}$	2.77	2.83	${\overset{¯}{X}}_{1}\neq {\overset{¯}{X}}_{2}$
a_e3_–a_e4_	19.00	1.09	$S_{1}^{2}=S_{2}^{2}$	2.77	1.59	${\overset{¯}{X}}_{1}={\overset{¯}{X}}_{2}$
a_e3_–a_e5_	19.00	2.42	$S_{1}^{2}=S_{2}^{2}$	2.77	0.93	${\overset{¯}{X}}_{1}={\overset{¯}{X}}_{2}$
a_e4_–a_e5_	19.00	3.17	$S_{1}^{2}=S_{2}^{2}$	2.77	−0.19	${\overset{¯}{X}}_{1}={\overset{¯}{X}}_{2}$

**Table 13:** Results of statistical analysis for a_e_ variable during milling from composite layer (CFRP/Al). [28].

**Compared Parameters**	**Fischer-Snedecor Test**			**Student’s t-Test**		
	**F Statistics Value**	**F_α_ Critical Value**	**Result**	**t Statistics Value**	**t_α_ Critical Value**	**Result**
a_e1_–a_e2_	1.87	19.00	$S_{1}^{2}=S_{2}^{2}$	−18.19	2.77	${\overset{¯}{X}}_{1}\neq {\overset{¯}{X}}_{2}$
a_e1_–a_e3_	1.27	19.00	$S_{1}^{2}=S_{2}^{2}$	11.30	2.77	${\overset{¯}{X}}_{1}\neq {\overset{¯}{X}}_{2}$
a_e1_–a_e4_	12.47	19.00	$S_{1}^{2}=S_{2}^{2}$	24.25	2.77	${\overset{¯}{X}}_{1}\neq {\overset{¯}{X}}_{2}$
a_e2_–a_e3_	2.37	19.00	$S_{1}^{2}=S_{2}^{2}$	29.44	2.77	${\overset{¯}{X}}_{1}\neq {\overset{¯}{X}}_{2}$
a_e2_–a_e4_	6.68	19.00	$S_{1}^{2}=S_{2}^{2}$	60.83	2.77	${\overset{¯}{X}}_{1}\neq {\overset{¯}{X}}_{2}$
a_e3_–a_e4_	15.84	19.00	$S_{1}^{2}=S_{2}^{2}$	7.04	2.77	${\overset{¯}{X}}_{1}\neq {\overset{¯}{X}}_{2}$
**Compared Parameters**	**Fischer-Snedecor Test**			**Cochran Cox-Test**		
	**F Statistics Value**	**F_α_ Critical Value**	**Result**	**C Statistics Value**	**C_α_ Critical Value**	**Result**
a_e1_–a_e5_	708.32	19.00	$S_{1}^{2}>S_{2}^{2}$	0.44	4.30	${\overset{¯}{X}}_{1}={\overset{¯}{X}}_{2}$
a_e2_–a_e5_	1321.83	19.00	$S_{1}^{2}>S_{2}^{2}$	0.35	4.30	${\overset{¯}{X}}_{1}={\overset{¯}{X}}_{2}$
a_e3_–a_e5_	557.71	19.00	$S_{1}^{2}>S_{2}^{2}$	1.56	4.30	${\overset{¯}{X}}_{1}={\overset{¯}{X}}_{2}$
a_e4_–a_e5_	8835.37	19.00	$S_{1}^{2}>S_{2}^{2}$	1.81	4.30	${\overset{¯}{X}}_{1}={\overset{¯}{X}}_{2}$

## References

[1] Oluwarotimi S., Nath H., Popov I., Beaugrand J. (2016). Comprehensive study on machinability of sustainable and conventional fibre reinforced polymer composites. Eng. Sci. Technol. Int. J..

[2] Nor A., Burbon W., Bert C. (1996). Computation models for sandwich panels and shells. Appl. Mech. Rev..

[3] Prasad D.S., Shoba C.H. (2014). Hybrid composites—A better choice for high wear resistance materials. J. Mater. Res. Technol..

[4] Mamalis M., Spentzas K., Pantelelis N., Manolakos D., Ioannidis M. (2008). A new hybrid concept for sandwich structures. Compos. Struct..

[5] Lu H., Wang X., Zhang T., Cheng Z., Fang Q. (2009). Design, fabrication, and properties of high damping metal matrix composites—A review. Materials.

[6] Botelho E.C., Pardini L.C., Rezende M.C. (2005). Hydrothermal, effects on damping behavior of metal/glass fiber/epoxy hybrid composites. Mater. Sci. Eng. A.

[7] Vamja D., Tejani G. (2013). Experimental test on sandwich panel. Composite material. IJIRSET.

[8] Icardi U., Ferrero L. (2009). Optimization of sandwich panels with functionally graded core and faces. Compos. Sci. Technol..

[9] Zhou D., Stronge W. (2005). Mechanical properties of fibrous core sandwich panels. Int. J. Mech. Sci..

[10] Arbaoui J., Schmitt Y., Pierrot J.L., Royer F.X. (2014). Effect of core thickness and intermediate layers on mechanical properties of polypropylene honey comb multilayer sandwich structures. Arch. Metall. Mater..

[11] Daniel I.M. (2009). Influence of core properties on the failure of composite sandwich beams. J. Mech. Mater. Struct..

[12] Hýsek Š., Frydrych M., Herclík M., Louda P., Fridrichová L., Le Van S., Le Chi H. (2019). Fire-Resistant Sandwich-Structured Composite Material Based on Alternative Materials and Its Physical and Mechanical Properties. Materials.

[13] Hýsek Š., Frydrych M., Herclík M., Fridrichová L., Louda P., Kníže R., Le Van S., Le Chi H. (2019). Permeable Water-Resistant Heat Insulation Panel Based on Recycled Materials and Its Physical and Mechanical Properties. Molecules.

[14] Feito N., Diaz-Alvarez J., Lopez-Puente J., Miguelez M.H. (2016). Nimerical analysis of the influence of tool wear and special cutting geometry when drilling woven CFRPs. Compos. Struct..

[15] Lopresto V., Caggiano A., Teti R. (2016). High Performance Cutting of Fibre Reinforced Plastic Composite Materials. Procedia CIRP.

[16] Yue X., Yang X., Tian J., He Z., Fan Y. (2018). Thermal, mechanical and chemical material removal mechanism of carbon fiber reinforced polymers in electrical discharge machining. Int. J. Mach. Tool Manuf..

[17] Zitoune R., Krishnaraj V., Collombet F. (2010). Study of drilling of composite material and aluminium stack. Compos. Struct..

[18] Zhang L., Liu Z., Tian W., Liao W. (2015). Experimental studies on the performance of different structure tools in drilling CFRP/Al alloy stacks. Int. J. Adv. Manuf. Technol..

[19] Nataraj M., Balasubramanian K. (2017). Parametric optimization of CNC turning process for hybrid metal matrix composite. Int. J. Adv. Manuf. Tech..

[20] Carlsson L.A., Kardomateas G.A. (2011). Structural and Failure Mechanics of Sandwich Composites.

[21] Kulkarni P. (2015). Evaluation of mechanical properties of Al 2024 based hybrid metal composites. J. Mech. Civ. Eng..

[22] PN-EN (2019). 573-3: 2019-12: Aluminum and Aluminum Alloys. Chemical Composition and Form of Wrought Products. Part 3: Chemical Composition and Form of Products.

[23] DIN EN (1997). 2563: Luft—und Raumfahrt—Kohlenstoffaserverstärkte Kunststoffe—Unidirektionale Laminate; Bestimmung der Scheinbaren Interlaminaren Scherfestigkeit.

[24] DIN ISO (2012). 527: Kunststoffe—Bestimmung der Zugeigenschaften—Teil 1: Allgemeine Grundsätze.

[25] DIN ISO (2011). 14125: Faserverstärkte Kunststoffe—Bestimmung der Biegeeigenschaften.

[26] Hoffmann Group (2019). Catalogue 1 Machining/Clamping Technology.

[27] Mishra P., Pandey C.M., Singh U., Gupta A., Sahu C., Keshri A. (2019). Descriptive statistics and normality tests for statistical data. Ann. Card. Anaesth..

[28] Benjamin D.J., Berger J.O., Johannesson M., Nosek B.A., Wagenmakers E.J., Berk R., Cesarini D. (2018). Redefine statistical significance. Nat. Hum. Behav..

[29] Slamin M., Gauthier S., Chatelain J.-F. (2014). Analysis of trajectory deviation during high speed trimming of carbon-fiber reinforced polymers. Robot. CIM-Int. Manuf..

[30] Uhlmann E., Richciarz S., Sammler F., Hufschmied R. (2016). High Speed Cutting of Carbon Fibre Reinforced Plastics. Procedia Manuf..

[31] Gara S., Tsoumarev O. (2016). Effect of tool geometry on surface roughness in slotting of CFRP. Int. J. Adv. Manuf. Technol..

[32] Azmi A.J., Lin R.J.T., Bhattacharyya D. (2013). Machinability study of glass fibre-reinforces polymer composites during end milling. Int. J. Adv. Manuf. Technol..

[33] Boudelier A., Ritou M., Garnier S., Furet B. (2011). Optimization of process parameters in CFRP machining with diamond abrasive cutters. Adv. Mater. Res..

